# Association between health literacy and mortality: a systematic review and meta-analysis

**DOI:** 10.1186/s13690-021-00648-7

**Published:** 2021-07-01

**Authors:** Zhao-ya Fan, Yuan Yang, Fan Zhang

**Affiliations:** 1 School of Public Health and Management, Chongqing Medical University, No.61 Daxuecheng Middle Road, Shapingba District, Chongqing, 400016 China; 2 Department of Cardiovascular Medicine, The First Affiliated Hospital of Chongqing Medical University, Chongqing, 400016 China

**Keywords:** Health literacy, Mortality, Correlation coefficient, Meta-analysis

## Abstract

**Background:**

To identify the relationship between health literacy (HL) and mortality based on a systematic review and meta-analysis.

**Methods:**

Literature published from database inception until July 2020 was searched using the PubMed and Web of Science databases, using relevant keywords and clear inclusion and exclusion criteria. The search was limited to English language articles. Two reviewers independently selected studies and extracted data. Pooled correlation coefficients and their 95% confidence intervals (CI) between HL and mortality were estimated using Stata 15.0 software. Potential sources of heterogeneity were explored using subgroup analysis, sensitivity analysis, and meta-regression. Quality of the original studies that were included in the meta-analysis was evaluated using the Newcastle–Ottawa Scale. A funnel plot and Egger’s test were used to determine whether significant publication bias was present.

**Results:**

Overall, 19 articles were included, reporting on a total of 41,149 subjects. Eleven were prospective cohort studies, and all articles were considered “good” quality. The most used screening instruments were the short Test of Functional Health Literacy (S-TOFHLA) in Adults and the Brief Health Literacy Screen (BHLS). Among 39,423 subjects (two articles did not report the number of patients with low HL), approximately 9202 (23%) had inadequate or marginal HL. The correlation coefficient between HL and mortality was 1.25 (95%CI = 0.25–0.44).

**Conclusion:**

Lower HL was associated with an increased risk of death. This finding should be considered carefully and confirmed by further research.

## Background

### Health literacy

Health literacy (HL) is the result of the healthcare and education system, and social, cultural, economic, and other factors. Improving HL is considered to be one of the most fundamental, economical, and effective measures to improve the health level of the whole population [1]. The Institute of Medicine defines HL as “the degree to which individuals can obtain, process, and understand the basic health information and services they need to make appropriate health decisions” [2]. It involves not only reading and understanding, but also the use of printed information, numerical information, and language literacy, etc. [3]. Patients without these skills cannot provide adequate self-care and may be at risk of higher mortality [4]. The 9th Global Conference on Health Promotion pointed out that HL is an important factor for ensuring improved health outcomes [5]. Low-level HL has long been regarded as a major obstacle to the effective management of cardiovascular diseases, affecting individual self-care skills and the health outcome of the patient, mainly in terms of doctor-patient communication, use of medical resources, quality of life, and mortality [6]. Inadequate HL has been linked with poor disease management, non-compliance with treatment recommendations, and medication errors by patients or caregivers. Whether for patients or medical workers, HL plays an irreplaceable role in disease prevention and management [7].

### Health literacy levels

At present, the status of HL in the world is not optimistic. The European Health Literacy Survey (HLS-EU) Consortium conducted a wide range of HL surveys in eight EU member states between 2009 and 2012, and the results showed that 47% of the 7770 respondents had limited (insufficient or problematic) HL [8]. According to the 2003 International Adult Literacy and Life Skills Survey (IALSS), more than 12 million (60%) adult Canadians lack HL; the overall level of HL in China in 2018 was 17% [9].

At the patient population level, adequate HL is the basis for disease prevention and management [10], but in fact HL of patients is not satisfactory. A meta analysis showed that among 13,457 patients with type 2 diabetes mellitus (T2DM), limited HL ranged from 7 to 82%, the lowest in Switzerland and the highest in Taiwan. Pooled prevalence showed nearly one-third patients with T2DM in the USA had limited functional HL [11]. Pooled prevalence of limited HL was 25% among patients with chronic kidney disease [12]. A cross-sectional study in France showed that the prevalence of low HL in patients with acute decompensated heart failure and acute myocardial infarction was 51 and 21%, respectively [13].

### Research justification

Understanding the impact of HL is a priority for health promotion, prevention and treatment of chronic diseases. Patients with inadequate HL have limited ability to obtain health information and understand disease-related knowledge, lack of correct cognition of disease, and are prone to negative emotions, which affect the treatment effect and lead to adverse outcomes [14]. Studies have shown that inadequate HL is associated with increased emergency use and readmission rates [15]. Many scholars have investigated the relationship between HL and mortality, although the results are inconsistent. McNaughton et al. investigated 1379 patients with acute heart failure and found that lower levels of HL were associated with an increased risk of death after hospitalization for acute heart failure [16]. However, León-González et al. conducted a prospective study of 556 patients with comorbid heart failure in six hospitals in Spain, and the results showed that there was no association between HL and 12-month mortality [17]. Therefore, the aim of this study was to provide a comprehensive analysis of the literature regarding the association between HL and mortality.

## Methods

We performed this review according to the recommendations of the Cochrane Collaboration and following the PRISMA Statement. The PROSPERO registration number is CRD42020203347.

### Search strategy

Relevant studies were identified through the Web of Science Core Collection and PubMed databases by using the following search terms, and the search strategy was specific to each database: 1) health literacy: “health literacy” OR “healthy literacy” OR “literacy”; 2) mortality: “mortality” OR “death” OR “fatal”. The searches were limited to full-length articles published in English, and the results were downloaded into Endnote X9.2 (Thomson Reuters (Scientific) LLC Philadelphia, PA). A more extensive and detailed search strategy is reported in Table 1.

**Table 1:** Search methods for identification of studies: A systematic review and meta-analysis on the association between HL and mortality from 2006 to 2020

Database	Search strategy	Results
Web of Science Core Collection	#1: TOPIC: (“health literacy”) OR TOPIC: (“healthy literacy”) OR TOPIC: (literacy)	17411
	#2: TOPIC: (mortality) OR TOPIC: (death) OR TOPIC: (fatal)	1397471
	#3: #1 AND #2	1170
PubMed	#1: ((health literacy [MeSH Terms]) OR (healthy literacy [MeSH Terms])) OR (literacy [MeSH Terms])	6558
	#2: ((mortality [MeSH Terms]) OR (death [MeSH Terms])) OR (fatal [MeSH Terms])	514466
	#3: #1 AND #2	65
	Total	1235

*MeSH* Medical Subject Headings

### Inclusion and exclusion criteria

Studies were selected if they met the following criteria: 1) the study assessed HL using a previously validated instrument; 2) the main outcome was death, including all-cause mortality and special mortality; and 3) the study assessed the correlation between HL and mortality, and provided hazard ratio (HR), relative risk (RR), or odds ratio (OR) estimates and corresponding 95% confidence intervals (CI). We excluded articles: 1) in languages other than English; 2) that were editorials, conference abstracts, letters, book news, or review articles; 3) in which HL or death was not measured; and 4) in which there was no correlation coefficient between HL and mortality provided. When more than one study reported results from the same cohort, the most recent and detailed studies were included in the analysis.

### Data collection

First, two reviewers (Zhaoya F and Yang Y) independently screened the articles by title and abstract. Then, the full-text was read and the remaining articles were filtered again. Any disagreements in the process were resolved by consensus.

For each study included in the systematic review, we extracted the following data using a standardized form: first author, year of publication, study design, geographic location, source population, baseline age and sex of participants, subject ethnicity, duration of follow-up, number of deaths, how HL was evaluated, HR RR or OR and the corresponding 95% CI, and adjustments for covariates.

### Quality assessment

The quality of the original studies that were included in the meta-analysis was evaluated using the Newcastle-Ottawa Scale [18]. The quality assessment scale awards 0–13 points based on three perspectives: selection of study population, comparability, and outcome assessment. We considered studies with a total score of ≥9 points to represent high quality. Scoring for quality assessment was independently conducted by two authors (Zhaoya F and Yang Y). Their results were compared and a third party (Zhang F) intervened if a consensus could not be reached.

### Statistical analysis

Most studies divided patients into two categories based on similar cutoff points: adequate and inadequate HL. When the results of adequate, marginal, and inadequate HL were presented, we combined the inadequate and marginal HL categories according to previous studies, which have shown that any inadequate HL is a risk factor for outcomes. HR was used to measure the association between HL and mortality. When studies had not used the highest category as a reference, we recalculated the HRs and their 95% CI relative to the highest category [19, 20]. For studies that separately calculated the relationship between inadequate and marginal HL and mortality, we combined the HRs using the method reported by Hamling and then used the pooled HRs for the overall meta-analysis [21]. To combine the S-TOFHLA with other measurement tools to evaluate HL, we chose the result of the S-TOFHLA assessment.

Inter-study heterogeneity was evaluated using Cochran’s χ^2^-based Q statistic, and inconsistency was quantified using the I^2^ statistic. I^2^ values of 0, 25, 50, and 75% were considered as no, low, moderate, and high degrees of heterogeneity, respectively [22]. According to the Q-statistic, if no significant heterogeneity (defined as I^2^ < 50%) was found, the pooled HR estimate was determined with the fixed effects model; the random effects model was used in the case of significant heterogeneity. Stratification analyses by population, study design, area, time, and the types of HL instruments were conducted as a way of addressing inter-study heterogeneity. Sensitivity analysis was performed to ensure the stability of the results. Meta-regression analysis was used to detect heterogeneity. The dependent variable of meta-regression was the correlation coefficient between HL and mortality, and population, study design, year of publication, and geographic location were independent variables. Publication bias was assessed using Egger’s test [23]. Stata version 15.0 (Stata Corporation, College Station, TX, USA) was used for all the statistical analyses, and a two-tailed *P* < 0.05 was assumed to be statistically significant.

## Results

**Fig. 1:**
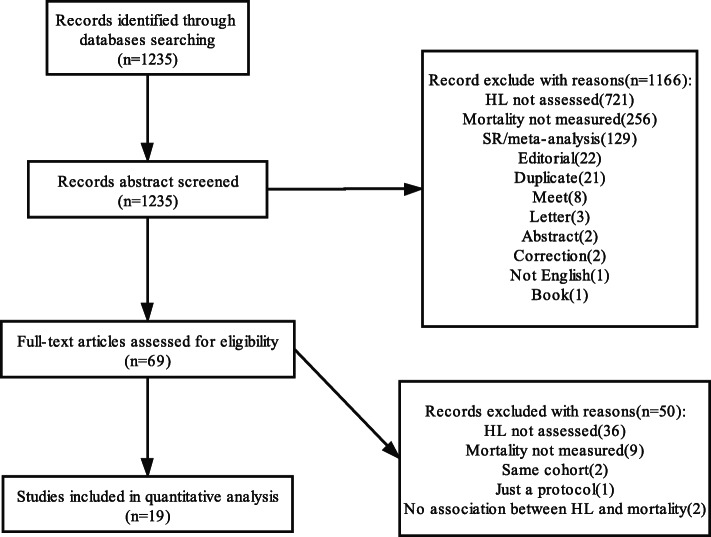
Flowchart of study selection: A systematic review and meta-analysis on the association between HL and mortality from 2006 to 2020

Initially, 1235 articles were identified. Sixty-nine were discarded after the first round screening of title and abstract. The main reason for exclusion was the failure to evaluate HL (*n* = 721) and mortality (*n* = 256). During the full-text review, 19 articles were selected and included in the systematic review. All the studies included were cohort studies. Specific reasons for exclusion and the selection procedure are shown in Fig. 1.

### Characteristics of included studies

The included studies were published between 2006 and 2020. Eleven were prospective cohort studies [17, 24–33] and eight were retrospective cohort studies [16, 34–40]. Among these articles, 41,149 participants were involved, of which 5840 (14%) died. The majority of the studies were undertaken in the United States [16, 34–40]. Four were carried out in the United Kingdom [27, 31, 34, 38], one in Spain [17], and one in Turkey [36]. Of the 19 studies, seven were conducted in the elderly [24, 27, 29, 30, 34, 38, 39], six in patients with heart failure [16, 17, 26, 33, 37, 40], one in chronic kidney disease patients [31], one in cardiovascular disease patients [28], one in chronic hemodialysis patients [25], one in kidney transplant candidates [32], one in palliative care patients [36], and one in male veterans [35]. The length of follow-up ranged from 3 months to 8 years (one study [31] did not provide data on follow-up period). The quality ratings of the studies are shown in Table 2. All studies were of high quality. Table 2 summarizes the characteristics of the included studies.

**Table 2:** Summary of the 19 publications included in a systematic review and meta-analysis on the association between HL and mortality from 2006 to 2020

Author	Location	Design	Study population	Sample size	Sex (% male)	Death	HL instrument	low HL	Study conclusion	Adjusted	NOS score
Baker et al. 2008 [24]	USA	prospective cohort	Elderly	3260	1388 (42.6%)	815	S-TOFHLA	1166	HR: 1.25 95%CI: 0.86–1.82	age, sex, race, education, income, language, SF-36 physical functioning, mental health component scores, number of chronic diseases, number of impairments in activities of daily living, number of impairments in instrumental activities of daily living, city of enrollment	9
Bostock et al. 2012 [34]	UK	retrospective cohort	Elderly	7857	3515 (44.7%)	621	4-item test	2579	HR: 1.15 95%CI: 0.79–1.67	age, sex, race, education, wealth, occupation, health status (limiting illness, limited activities of daily living, depressive symptoms, self reported doctor diagnosed disease: heart disease, diabetes, stroke, cancer, asthma, chronic lung disease), health behaviors (smoking, exercise, alcohol consumption), cognitive (orientation in time, immediate recall of word list, fluency in an animal naming task)	12
Cavanaugh et al. 2010 [25]	USA	prospective cohort	Hemodialysis patients	480	269 (56.0%)	103	REALM	154	HR: 1.51 95%CI: 0.99–2.30	age, sex, race, diabetes status, baseline serum albumin.	11
Fabbri et al. 2018 [26]	USA	prospective cohort	HF	2487	1333 (53.6%)	250	BHLS	261	HR: 1.91 95%CI: 1.38–2.65	age, sex, education, marital status, CCI	12
Fawns-Ritchie et al. 2018 [27]	UK	prospective cohort	Elderly	795	336 (42.3%)	130	S-TOFHLA、NVS REALM General functional health literacy	38 ± 3.85 score	HR: 1.00 95%CI: 0.95–1.05	age, sex, education, age-11 IQ, current fluid ability in older age, occupational class, health status	10
Ferri-Guerra et al. 2020 [35]	USA	retrospective cohort	Veterans	470	470 (100%)	63	NVS	258	HR: 1.08 95%CI: 0.95–1.22	age, race, educational, median household income, CCI, hospitalizations in the previous year	12
León-González et al. 2018 [17]	Spain	prospective cohort	HF	556	210 (37.5%)	189	SAHLSA	370	HR: 1.05 95%CI: 0.65–1.68	age, sex, education, treatment group (intervention, no intervention), hospital, NYHA, LVEF, atrial fibrillation, serum hemoglobin, serum creatinine, depression, CCI, Katz index, Mini-Mental State Examination, hospitalization for HF in last year, HF knowledge score, HF self-care score.	11
Mayberry et al. 2018 [28]	USA	prospective cohort	CVD	2977	1602 (53.8%)	304	BHLS S-TOFHLA、SNS-3	506	OR: 1.31 95%CI: 1.01–1.69	age, sex, race, socioeconomic status (employed, disabled, financial strain, income, underinsured)	9
McNaughton et al. 2015 [16]	USA	retrospective cohort	HF	1379	813 (59.0%)	403	BHLS	324	HR: 1.32 95%CI: 1.05–1.66	age, sex, race, education, insurance, index hospitalization length of stay, disease severity	10
Metin et al. 2019 [36]	Turkey	retrospective cohort	Palliative care patients	242	–	147	HLS-EU	195	HR: 1.44 95%CI: 0.90–2.32	age, height, weight, tracheostomy presence, triceps circumference, food intake status of the patients	10
Peterson et al. 2011 [37]	USA	retrospective cohort	HF	1494	699 (46.8%)	124	BHLS	262	HR: 1.04 95%CI: 0.79–1.37	age, sex, race, education, socioeconomic status, self-reported physical function, residential status (independent vs. nursing facility or hospice care), serum creatinine, LVEF, year of cohort entry	11
Smith et al. 2018 [38]	UK	retrospective cohort	Elderly	7731	3460 (44.8%)	1259	4-item test	959	HR: 1.22 95%CI: 1.03–1.46	age, sex, race, education, occupational class, wealth quintile, health status (limiting illness, functional impairment, depressive, self-reported doctor-diagnosed disease: heart disease, diabetes, cancer, stroke, asthma, chronic lung disease), health behaviors (smoke, exercise,alcohol consumption), cognitive (time orientation, recall of word list, fluency on an animal naming task), social isolation	10
Stewart et al. 2020 [29]	USA	prospective cohort	Elderly	931	158 (17.0%)	224	9-item instrument	65.8% correct	HR: 1.01 95%CI: 1.00–1.02	age, sex, education, income, lower total, health, financial literacy, medical conditions, physical activity, depressive symptoms, cognitive	11
Sudore et al. 2006 [30]	USA	prospective cohort	Elderly	2512	1225 (48.8%)	320	REALM	595	HR: 1.75 95%CI: 1.27–2.41	age, sex, race, education, income, health status (self-rated health, cardiac disease, stoke, cancer, hypertension, diabetes, obesity), health behaviors (smoke, alcohol consumption), health care access measures, psychosocial status	9
Taylor et al. 2019 [31]	UK	prospective cohort	CKD	2274	1474 (64.8%)	338	BHLS	364	HR: 1.05 95%CI: 0.73–1.49	age, sex, race, education, language, primary renal diagnosis, CCI, car ownership	9
Warsame et al. 2019 [32]	USA	prospective cohort	KT	1578	1205 (76.4%)	54	BHLS	140	HR: 2.42 95%CI: 1.16–5.05	age, sex, race, cause of end-stage renal disease, blood type	9
Wolf et al. 2010 [39]	USA	retrospective cohort	Elderly	2956	–	417	S-TOFHLA	643	HR: 1.95 95%CI: 1.37–2.77	age, sex, race, education, income, occupational class, number of chronic conditions, physical functioning, activity limitations, mental health	12
Wu et al. 2013 [33]	USA	prospective cohort	HF	595	309 (51.9%)	16	S-TOFHLA	220	RR: 1.31 95%CI: 1.06–1.63	age, sex, race, education,income, site, insurance, systolic blood pressure, systolic dysfunction, NYHA, diabetes, hypertension, atrial fibrillation, history of CVD (MI or angina), beta-blocker use, HF symptoms, HF general knowledge, salt knowledge, self-efficacy, self-care behaviors HF	12
Wu et al. 2016 [40]	USA	retrospective cohort	HF	575	341 (59.3%)	63	S-TOFHLA	206	HR: 1.80 95%CI: 1.29–2.50	age, sex, race, income, marital status, employed, LVEF, NYHA, BNP, ACEI user, beta-blocker user, intervention group	10

*ACEI* angiotensin-converting enzyme inhibitor, *BHLS* the Brief Health Literacy Screen, *BNP* B-natriuretic peptide, *CCI* Charlson Comorbidity Index, *CI* confidence interval, *CKD* chronic kidney disease, *CVD* chronic kidney disease, *HF* heart failure, *HLS-EU* Health Literacy Survey-European Union-Questionnaire, *HR* hazard ratio, *KT* kidney transplant, *LVEF* left ventricular ejection fraction, *MI* myocardial infarction, *NVS* the Newest Vital Sign, *NYHA* New York Heart Association, *REALM* the Rapid Estimate of Adult Literacy in Medicine, *SAHLSA* the Short Assessment of Health Literacy for Spanish-speaking Adults, *SNS-3* 3-item version of the Subjective Numeracy Scale, *S-TOFHLA* the short Test of Functional Health Literacy in Adults

### HL screening instruments

Nine different instruments were used to screen HL in the studies included in this systematic review: the S-TOFHLA [24, 27, 28, 33, 39, 40], Rapid Estimate of Adult Literacy in Medicine (REALM) [25, 27, 30], Brief Health Literacy Screen (BHLS) [16, 26, 28, 31, 32, 37], Newest Vital Sign [35], Short Assessment of Health Literacy for Spanish-speaking Adults [17], 3-item version of the Subjective Numeracy Scale [28], Health Literacy Survey-European Union-Questionnaire [36], a brief 4-item comprehension test based on instructions similar to those found on a packet of aspirin bought over the counter [38], general functional HL [27] and a 9-item instrument [29]. Two articles [27, 28] used more than one tool to measure HL. The most commonly used HL screening instrument was the S-TOFHLA and the BHLS, used in six studies.

The S-TOFHLA is a shortened version of the Test of Functional Health Literacy in Adults that includes two reading passages (36 items worth 2 points each) and four numeracy items (7 points each) [41]. This test is an objective test in which respondents choose words missing from text representing medical directions and information about health care, and the sum of the two parts yields the S-TOFHLA score, ranging from 0 to 100. The reading comprehension part of this test is mainly reading materials in the hospital environment, such as informed consent and label of a medicine bottle, and a calculation part assessing the patient’s numerical comprehension ability such as understanding blood glucose measurement values and financial subsidies. The S-TOFHLA divides respondents into three categories depending on scores: 0–55, 56–66, and 67–100, corresponding to inadequate literacy, marginal literacy, and adequate literacy, respectively.

The BHLS is a subjective measure, which consists of three items, asking patients to report their level of confidence filling out medical forms, need for assistance in reading hospital materials, and understanding of written medical information [42]. The specific questions are: 1) “How often do you have someone help you read hospital materials?”, 2) “How often do you have problems learning about your medical condition because of difficulty reading hospital materials?”, and 3) “How confident are you filling out forms by yourself?” Each question was scored by patients on a 5-point scale, in which higher scores indicated lower literacy. Compared with S-TOFHLA, the brief screener is less time-consuming and easier to implement in clinical practice.

The REALM is a word recognition and pronunciation test based on the correct pronunciation of 66 common medical terms designed for use in health care settings. The original REALM was developed in 1991 by Davis and consisted of 125 common medical terms [43]. The test format was revised in 1993, and the list of words was shortened to 66 items [44]. Participants are presented a piece of paper with a list of 66 medical words and are asked to read these words aloud. The words range in difficulty from easy (‘fat’) to difficult (‘impetigo’). One point is given for each correct response. A score of 59 or less is defined as indicating low HL, while a score of 60 or more indicates adequate HL. Many derivative versions have been developed to meet different needs. Lee et al. developed the Short Assessment of Health Literacy for Spanish-speaking Adults for the Spanish-speaking language group [45].

### HL and mortality

#### Overall analysis

**Fig. 2:**
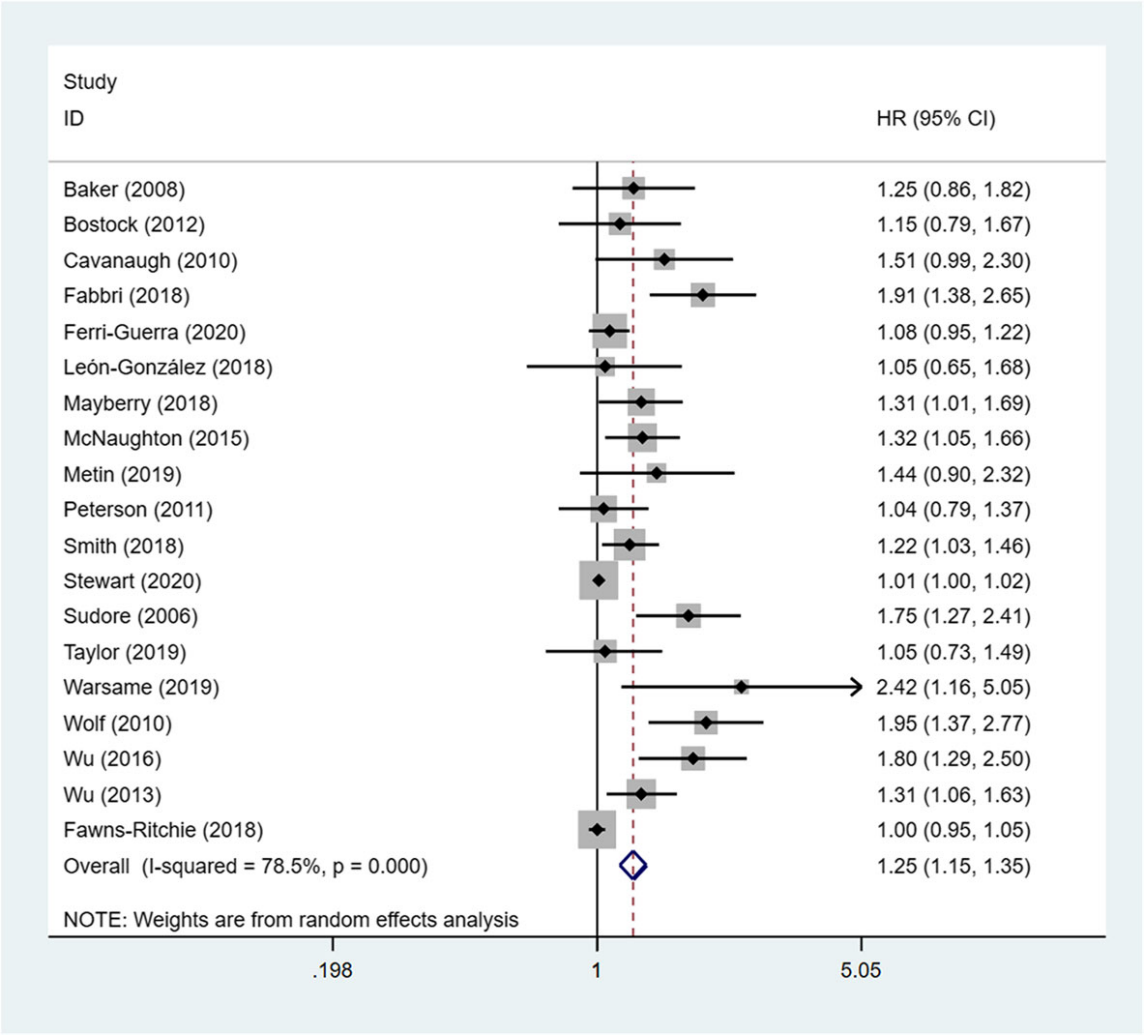
Forest plot of HL and risk of death: A systematic review and meta-analysis on the association between HL and mortality from 2006 to 2020

In the heterogeneity test, the correlation between HL and mortality (I^2^ = 78.5%, *P* < 0.001) showed that there was heterogeneity, using a random effects model to combine effect quantity. Based on the combined results of the 19 cohort studies, compared with the adequate category, inadequate or marginal categories experienced significantly increased risk of death (HR = 1.25, 95% CI =1.15–1.35) (Fig. 2).

#### Subgroup analysis

The subgroup analysis included population, study design, area, time, and the types of HL instruments (Table 3). For studies conducted in patients with heart failure (HF), the meta-analysis revealed a significantly increased risk of death among inadequate or marginal HL categories as compared to the adequate HL category (HR = 1.37, 95% CI = 1.14–1.65; *P* < 0.001; I^2^ = 57.9%). Noticeably, the correlation coefficient between HL and mortality was 1.20 (95% CI = 1.09–1.32; *P* < 0.001; I^2^ = 78.1%) based on the prospective cohort study design and 1.38 (95% CI = 1.20–1.59; *P* < 0.001; I^2^ = 84.3%) in the USA.

**Table 3:** Subgroup analysis of a systematic review and meta-analysis on the association between HL and mortality from 2006 to 2020

Subgroup	No. of studies	HR(95%CI)	*P*	I^2^
Total	19	1.25 (1.15–1.35)	< 0.001	78.5%
Population				
Elderly	7	1.14 (1.04–1.25)	< 0.001	80.6%
Heart failure	6	1.37 (1.14–1.65)	0.036	57.9%
Design				
Prospective	11	1.20 (1.09–1.32)	< 0.001	78.1%
Retrospective	8	1.30 (1.12–1.50)	0.012	61.3%
Location				
USA	13	1.38 (1.20–1.59)	< 0.001	84.3%
UK	4	1.07 (0.95–1.21)	0.167	40.7%
Time				
Before 2016.12	9	1.40 (1.22–1.61)	0.008	43.2%
After 2017.1	10	1.11 (1.03–1.20)	< 0.001	71.7%
HL questionnaire				
Model 1				
S-TOFHLA*	5	1.38 (1.05–1.83)	< 0.001	86.9%
BHLS	5	1.36 (1.05–1.77)	0.017	66.6%
REALM	2	1.66 (1.28–2.14)	0.585	0.0%
NVS	1	1.08 (0.95–1.22)	–	–
Others	6	1.14 (1.00–1.31)	0.051	54.6%
Model 2				
S-TOFHLA	4	1.52 (1.23–1.89)	0.126	47.5%
BHLS	5	1.36 (1.05–1.77)	0.017	66.6%
REALM*	3	1.35 (0.89–2.03)	0.001	85.9%
NVS	1	1.08 (0.95–1.22)	–	–
Others	6	1.14 (1.00–1.31)	0.051	54.6%
Model 3				
S-TOFHLA	4	1.52 (1.23–1.89)	0.126	47.5%
BHLS	5	1.36 (1.05–1.77)	0.017	66.6%
REALM	2	1.66 (1.28–2.14)	0.585	0.0%
NVS*	2	1.02 (0.91–1.14)	0.182	43.7%
Others	6	1.14 (1.00–1.31)	0.051	54.6%
Model 4				
S-TOFHLA	4	1.52 (1.23–1.89)	0.126	47.5%
BHLS	5	1.36 (1.05–1.77)	0.017	66.6%
REALM	2	1.66 (1.28–2.14)	0.585	0.0%
NVS	1	1.08 (0.95–1.22)	–	–
Others*	7	1.11 (0.99–1.24)	0.082	46.5%

*represent the tool used in the analysis

One study assessed HL using four separate tools, and each provided an association between HL and mortality. When subgroup analysis was conducted according to HL instruments, we used different models for analysis. The asterisk indicates the different tools used in the study, and we chose the results of S-TOFHLA assessment in other subgroup analyses.

#### Sensitivity analysis and meta-regression analysis

Sensitivity analyses were used to evaluate the effect of each study on the pooled results by sequentially excluding single studies. The results did not significantly change after excluding each study (Table 4).

**Table 4:** Sensitivity analysis of a systematic review and meta-analysis on the association between HL and mortality from 2006 to 2020

Omitting study	HR	95%CI	*P*	I^2^
Baker et al. 2008 [24]	1.25	1.15–1.35	< 0.001	79.4%
Bostock et al. 2012 [34]	1.25	1.15–1.36	< 0.001	79.6%
Cavanaugh et al. 2010 [25]	1.24	1.14–1.34	< 0.001	78.8%
Fabbri et al. 2018 [26]	1.21	1.12–1.31	< 0.001	75.4%
Fawns-Ritchie et al. 2018 [27]	1.32	1.18–1.48	< 0.001	79.6%
Ferri-Guerra et al. 2020 [35]	1.27	1.16–1.39	< 0.001	79.4%
León-González et al. 2018 [17]	1.25	1.15–1.36	< 0.001	79.7%
Mayberry et al. 2018 [28]	1.24	1.14–1.35	< 0.001	78.7%
McNaughton et al. 2015 [16]	1.24	1.14–1.35	< 0.001	78.3%
Metin et al. 2019 [36]	1.24	1.14–1.35	< 0.001	79.1%
Peterson et al. 2011 [37]	1.26	1.16–1.37	< 0.001	79.7%
Smith et al. 2018 [38]	1.25	1.15–1.36	< 0.001	78.6%
Stewart et al. 2020 [29]	1.32	1.18–1.47	< 0.001	75.1%
Sudore et al. 2006 [30]	1.22	1.13–1.32	< 0.001	76.5%
Taylor et al. 2019 [31]	1.26	1.16–1.36	< 0.001	79.7%
Warsame et al. 2019 [32]	1.23	1.14–1.34	< 0.001	78.3%
Wolf et al. 2010 [39]	1.22	1.12–1.31	< 0.001	75.8%
Wu et al. 2013 [33]	1.22	1.13–1.32	< 0.001	76.4%
Wu et al. 2016 [40]	1.24	1.14–1.35	< 0.001	78.3%

To identify the possible sources of heterogeneity, different factors associated with heterogeneity, such as population, study design, year of publication, and geographic location, were computed using meta-regression models, although none of these variables were statistically significant.

#### Publication bias

**Fig. 3:**
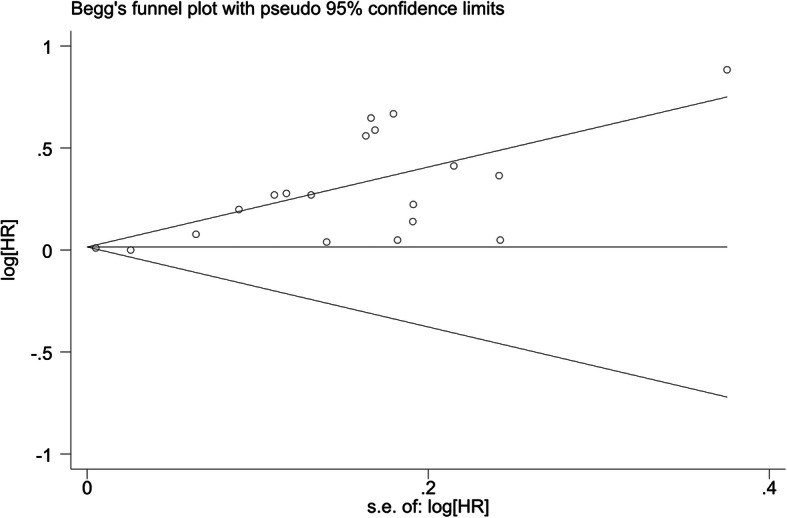
Funnel plot of a systematic review and meta-analysis on the association between HL and mortality from 2006 to 2020

**Fig. 4:**
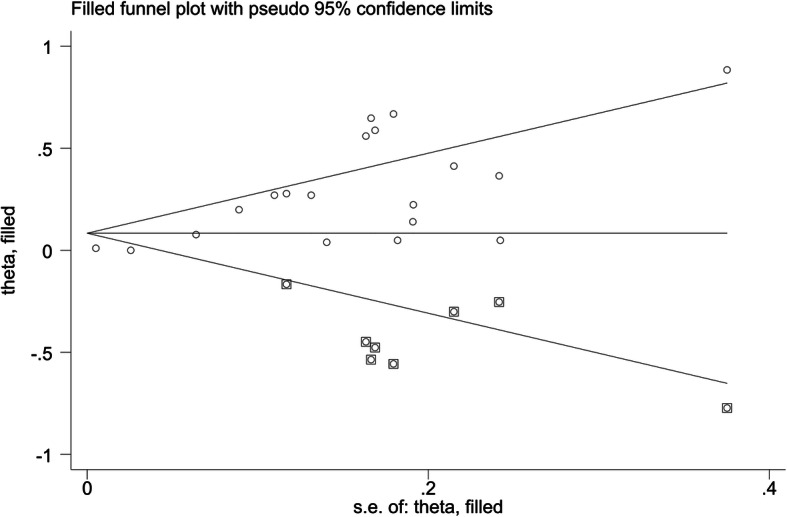
Trim and fill method for evaluating publication bias (The circles alone are real studies and the circles enclosed in boxes are ‘filled’ studies. The horizontal line represents the summary effect estimates, and the diagonal lines represent pseudo-95%CI limits): A systematic review and meta-analysis on the association between HL and mortality from 2006 to 2020

The publication bias test indicated significant publication bias. A funnel plot (Fig. 3) showed visual evidence of asymmetry, which was consistent with Egger’s regression symmetry test (*P* < 0.001), and we adjusted for the effect of publication bias by using the Duval and Tweedie’s nonparametric trim-and-fill method, which imputes hypothetical small missing null or negative studies [46]. After imputing eight missing studies, a symmetrical funnel plot was obtained (Fig. 4).

## Discussion

Low HL has important implications for wellness, increasing the risk of negative health outcomes, and is also an invisible barrier to health care services that has profound costs for individual and public health. With the development of medical technology and the increase in life expectancy, people pay more attention to their level of HL. Most researchers believe that HL is an important predictor of health status (even stronger than income, career, and education) [47]. Therefore, it is important to pay attention to improving HL in the population. The first step in overcoming the impact of low HL in the population is to recognize the high prevalence of limited HL [48]. For example, during hospital visits, surgeons should seek to enhance patient understanding, avoid using technical medical terminology, and encourage patients to participate in care discussions.

This study is a systematic review and meta-analysis of HL levels in the whole population. Among 39,423 subjects (two articles [27, 29] did not report the number of low HL), approximately 9202 (23%) had inadequate or marginal HL. In other studies, a similar conclusion was reached. Paasche-Orlowl et al. systematically reviewed the USA studies and examined the prevalence of limited HL; 31,129 subjects were involved, and a low prevalence of HL between 0 and 68% was reported. Pooled analyses of these data revealed that the weighted prevalence of low HL was 26% and that of marginal HL was 20% [49]. For six studies conducted in HF patient samples, the prevalence of inadequate or marginal HL was 17%. In a previous systematic review conducted by Fabbri et al. [50], it was found that an average of 24% of HF patients had inadequate or marginal HL. Our result was slightly lower than this.

Previous studies have shown that the most common demographic features reported to be associated with HL are age, ethnicity, and geographic location [49]. Our study showed that inadequate HL was associated with a higher risk of mortality. In contrast, three articles did not find an association between HL and mortality. One study was conducted only in male veterans [35], one was conducted in Spain in HF patients [17], and one was conducted in the UK in chronic kidney disease patients [31]. These may be the reasons for the different conclusions, reflecting two factors: different care delivery systems may be a factor in the outcome, underscoring the need for further studies to be conducted in different countries; and mortality has high statistical heterogeneity, which may be caused by the different populations in the studies.

Nine different instruments were used to screen HL in the studies included in this systematic review. Instruments vary in how they transform the concept of HL into a measurable construct. Most measures involve only limited conceptual dimensions of HL. The time and resources required to implement measures vary considerably across the measures. Scoring approaches and categories of HL on the basis of performance measures also vary. It is worth noting that the studies included in this review conceptually defined HL in a variety of ways. Five of the studies [16, 24, 30, 31, 34] failed to provide a conceptual definition of HL; 12 of the studies [17, 25–27, 29, 32, 34, 36–40] simply defined HL as “the degree to which individuals have the capacity to obtain, process, and understand basic health information and services needed to make basic health decisions”, failing to recognize the multifaceted nature of HL that goes beyond these abilities. The rest [28, 35] recognized HL as a multidimensional process, incorporating systemic demands and complexities as well as individuals’ skills and abilities, and may encompass numerical and graphical literacy. The differences in the conceptual definitions provided are not surprising given that there is no universal consensus on the definition of HL. Regardless of differing opinions, most experts agree that HL is more than just the ability to read and comprehend health information. At the same time, among the population surveyed, the pooled estimate might overestimate the actual prevalence of low HL. For studies where most of the subjects were patients, they excluded patients who could not speak or understand English, and those with cognitive impairment. In addition, the studies required signed informed consent, which could have discouraged patients with low HL from participating in the studies, given that most consent forms are written at a 10th-grade reading level. Various interventions and screening instruments, as well as the variety of outcome parameters across many time periods, mitigated the use of meta-analysis, so caution should be taken when interpreting the findings presented in this review.

Due to the heterogeneity observed among the included studies, pooled estimates were calculated using the random effects model for both the overall analysis and for several of the subgroup analyses. This model assumes that the underlying true effects differ between studies. Sources of heterogeneity could include differences in participant characteristics across studies, study design factors, and variations in the metrics (RR versus HR) used to measure outcomes. For the present study, using sensitivity analysis, no study was found to significantly contribute to the heterogeneity.

Finally, publication bias was detected; the funnel plot revealed an apparent asymmetry that suggested the presence of a potential publication bias, a language bias, inflated estimates by a flawed method logic design in smaller studies, and/or a lack of publication of small trials with opposite results.

### Study strengths and limitations

The strengths of this study that lend weight to our conclusions are the large sample size and the use of validated literacy assessment instruments in almost all studies. The results of the present analysis are intended to provide more robust evidence than any individual study. However, some limitations may have influenced the findings, in that heterogeneity was observed among the included studies and publication bias could not be avoided. Second, the included studies assessed levels of HL with different tools. Although we conducted a stratified analysis based on the type of instrument, it may still affect comparability because subjective and objective measurement tools may have different focuses. Finally, due to the author’s inability to review non-English manuscripts, only English articles were included in this study, which may result in the loss of some studies.

### Suggestions for further studies

Most of the studies were conducted in the USA, which limits the generalizability of the findings to other countries with different healthcare systems and social structures. Future research should be conducted in different countries and regions to increase the generalizability. Second, future studies should consider the use of a more complete measure of HL, one that measures all the dimensions of HL and not only reading comprehension.

## Conclusions

The prevalence of low HL ranged from 9 to 81% (two articles [27, 29] did not report the number of low HL), with an average of 23% of the study participants found to have low HL. This meta-analysis suggests that HL is associated with mortality. However, this conclusion needs to be supported by further evidence. Considering the increasing prevalence of inadequate HL worldwide and the heavy burdens of death, it is essential to simplify health services and improve health education. Our findings may provide valuable clues for related research in the future.

## Data Availability

All relevant data are within the paper and its Supporting Information files.

## Abbreviations

BHLS: the Brief Health Literacy Screen
CI: Confidence Interval
HL: Health Literacy
HR: Hazard Ratio
OR: Odds Ratio
REALM: the Rapid Estimate of Adult Literacy in Medicine
RR: Relative Risk
S-TOFHLA: the Short Test of Functional Health Literacy in Adults
